# Cancer physicians’ attitude towards treatment of the elderly cancer patient in a developed Asian country

**DOI:** 10.1186/1471-2318-13-35

**Published:** 2013-04-16

**Authors:** Angela Pang, Shirlynn Ho, Soo-Chin Lee

**Affiliations:** 1 Department of Haematology-Oncology, National University Cancer Institute, National University Health System, Level 7, Tower Block, 1E, Kent Ridge Road, Singapore, 119228, Singapore; 2 Department of Palliative Medicine, National Cancer Centre, Singapore, Singapore; 3 Cancer Science Institute, National University of Singapore, Singapore, Singapore

**Keywords:** Geriatric oncology, Geriatric oncology service, Cancer disclosure, Cancer treatment, Cancer physicians

## Abstract

**Background:**

With an aging population and an increasing number of elderly patients with cancer, it is essential for us to understand how cancer physicians approach the management and treatment of elderly cancer patients as well as their methods of cancer diagnosis disclosure to older versus younger patients in Singapore, where routine geriatric oncology service is not available.

**Methods:**

57 cancer physicians who are currently practicing in Singapore participated in a written questionnaire survey on attitudes towards management of the elderly cancer patient, which included 2 hypothetical clinical scenarios on treatment choices for a fit elderly patient versus that for a younger patient.

**Results:**

The participants comprised of 68% medical oncologists, 18% radiation oncologists, and 14% haematologists. Most physicians (53%) listed performance status (PS) as the top single factor affecting their treatment decision, followed by cancer type (23%) and patient’s decision (11%). The top 5 factors were PS (95%), co-morbidities (75%), cancer stage (75%), cancer type (75%), patient’s decision (53%), and age (51%). 72% of physicians were less likely to treat a fit but older patient aggressively; 53% and 79% opted for less intensive treatments for older patients in two clinical scenarios of lymphoma and early breast cancer, respectively. 37% of physicians acknowledged that elderly cancer patients were generally under-treated.

Only 9% of physicians chose to disclose cancer diagnosis directly to the older patient compared to 61% of physicians to a younger patient, citing family preference as the main reason. Most participants (61%) have never engaged a geriatrician’s help in treatment decisions, although the majority (90%) would welcome the introduction of a geriatric oncology programme.

**Conclusions:**

Advanced patient age has a significant impact on the cancer physician’s treatment decision-making process in Singapore. Many physicians still accede to family members’ request and practice non-disclosure of cancer diagnosis to geriatric patients, which may pose as a hurdle to making an informed decision regarding management for the geriatric cancer patients. Having a formal geriatric oncology programme in Singapore could potentially help to optimize the management of geriatric oncology patients.

## Background

With an ageing population coupled with healthcare advances, it is expected that an increasing proportion of patients diagnosed with cancer will be above the age of 65 years worldwide including Asia [1]. Ageing is associated with an accumulation of medical and social problems as well as a reduction in physiologic reserves. These issues need to be taken into account before initiating elderly patients on cancer treatment [2] as they may face greater treatment related toxicities [3,4].

Singapore is a high-income country within Southeast Asia with developed healthcare services. While geriatrics medicine has been an established specialty in Singapore for 25 years in Singapore, there are as yet no formal geriatric oncology services in Singapore and no standardized approach or guidelines in the decision making process in the treatment of elderly cancer patients. There are 160 registered cancer specialists in Singapore, including 80 medical oncologists, 37 radiation oncologists and 43 haematologists as of 2011. The number of new cancer patients are on the rise in Singapore, with 11,069 patients diagnosed with cancers in year 2010 compared to 9,417 in 2006 [5]. Patients aged 70 years or older make up approximately 40% of the cancer patient load in Singapore, [6] but there are no registered geriatric oncologists in Singapore.

As older patients were often excluded from cancer clinical trials based on age exclusion criteria [7], the optimal approach to treatment options for these patients are often unclear. However, retrospective data and observational studies have suggested that many older adults can indeed tolerate and benefit from intensive cancer treatments, [8-12] and several geriatric assessment scales have been developed to standardize the decision making process, [13-20] although these scales are still not routinely used by most cancer physicians in Singapore.

In Singapore and many Asian countries, most cancer physicians continue to be deterred by the patients’ advanced age alone and opt for less intensive therapies which are often also less optimal options [21-23]. A recent report showed that older Asian breast cancer patients presented with more advanced disease but were less likely to receive standard treatment [21]. As cancer is still largely considered a taboo in Asia with many having a fatalistic view towards cancer in Asia, another potential barrier to optimal management is the reluctance to disclose cancer diagnosis to the elderly patient. Understanding cancer physicians’ attitudes towards treating older patients is key to developing strategies to improve cancer care for these patients. There have been several surveys with varying sample sizes and survey return rates conducted in other countries to evaluate this [24-27] but none to date in Singapore.

We devised a questionnaire to survey the cancer physicians in Singapore to identify factors that affect physicians’ decision making process prior to initiating treatment for older patients. In addition, we investigated cancer physicians’ preferences when dealing with the issue of cancer diagnosis disclosure to elderly patients, and explored the level of understanding and acceptance of the introduction of a multi-disciplinary geriatric oncology team in the management of elderly cancer patients.

## Methods

### Participants

The Singapore Medical Council Register of Medical Practitioners was used to identify cancer physicians in the respective specialties (medical oncology, radiation oncology, malignant haematology). In 2011, there were 80 fully registered medical oncologists, 37 radiation oncologists and 43 haematologists in Singapore. All cancer physicians who were actively providing care for cancer patients were invited to participate in the questionnaire survey which were distributed to the physicians in person during combined cancer seminars and collected back from responding physicians in the same setting. 150 questionnaires were distributed between April to October 2011. The study protocol was approved by the local institutional ethics review board. Waiver of written informed consent was granted as the identity of the responding physicians was not collected, and return of completed questionnaire constituted implied consent.

### Questionnaire format

We devised our questionnaire survey after reviewing the relevant literature on similar surveys that have been conducted amongst cancer physicians [24-27]. The survey was pilot tested in our institution amongst 10 cancer physicians who had at least 8 years of experience treating cancer patients to assess the response rates; feedback was collected with regards to the validity of the questionnaire. Minor modifications to the survey were made following the pilot study. The survey (Additional file 1) consisted of a segment on demographics of the participants, 11 multiple-choice questions, and 2 clinical scenarios.

In the 11 multiple choice questions, participants were asked to rank the most important single factor as well as the top 5 factors amongst 15 common factors that were likely to affect the decision making process with regards to treatment for the elderly oncology patients. The survey also explored cancer physicians’ perceptions of the influence of patients’ chronological age on their treatment choices. In addition, the topic of disclosure of cancer diagnosis to older patients as compared to younger patients was evaluated. The level of understanding and exposure to current geriatric assessment scales and potential acceptance of a multidisciplinary geriatric team into the local practice was explored.

Two case scenarios were used to evaluate the current practice patterns of cancer physicians in Singapore in managing elderly patients. In the hypothetical scenarios, physicians were asked to choose the most appropriate treatment regimen for an older patient and a younger patient with the same medical condition. The first case scenario described a fit elderly patient in otherwise good health who was diagnosed with stage IV diffuse large B cell non-Hodgkin’s lymphoma (DLBCL). The participants were asked to choose amongst various curative and palliative treatment options. The second scenario described a geriatric patient who had undergone breast lumpectomy for stage IIB, high risk node positive, hormone receptor positive, breast cancer. The participants were given treatment options that included adjuvant endocrine therapy alone and various chemotherapy regimens combined with endocrine therapy, which would be offered to younger patients to reduce the risk of disease recurrence and to improve overall survival. As adjuvant radiation therapy is the standard of care for women who had undergone lumpectomy, the participants were also asked if they would administer radiation therapy to the elderly patient.

## Results

### Participant demographics (Table 1)

**Table 1:** Participants’ Demographics (n = 57)

**Characteristics**	**Number**	**Percentage**
**Gender**		
Male	36	63%
Female	21	37%
**Age**		
21–30	9	16%
31–40	35	61%
41–50	11	19%
51–60	2	4%
**Specialty**		
Haematologist	8	14%
Medical Oncologist	39	68%
Radiation Oncologist	10	18%
**Institution**		
Public	51	89%
Private	6	11%
**Number of years in cancer practice**		
1–5 yrs	37	65%
6–10 yrs	7	12%
>10 yrs	13	23%
**Average number of patients per week**		
5–25	17	30%
26–50	27	47%
51–70	5	9%
71–99	4	7%
≥100	4	7%
**Percentage of patients > 65 years old**		
15–30%	13	23%
31–60%	33	58%
61–70%	11	19%
71–100%	0	0%

Of the 150 questionnaires distributed to cancer physicians, fifty-seven responded with a return rate of 38%. The majority were medical oncologists (68%), followed by radiation oncologists (18%) and haematologists (14%). Most practiced in public hospitals (90%), while 10% were in private practice. The majority of the participants were male (63%). About one-third of the participants had been in cancer practice for 6 years or more and 70% treated more than 25 patients per week, typically divided over 2–3 clinic sessions lasting 3–4 hours each. 77% of participants reported that geriatric patients older than 65 years made up more than 30% of their patient population.

### Factors influencing physicians’ decision for treatment of the geriatric oncology patient (Table 2)

**Table 2:** Factors affecting physicians’ decision for treatment of geriatric patients

**Factor**	**Number of physicians listing as the most important single factor**	**Percentage**
Performance status	30	53%
Type of Cancer	13	23%
Patient’s decision	6	11%
Age	3	5%
**Factor**	**Number of physicians listing as one of the top 5 factors**	**Percentage**
Performance status	54	95%
Co-morbidities	43	75%
Type of Cancer	43	75%
Stage of Cancer	43	75%
Patient’s decision	30	53%
Age	29	51%
**Will physicians be less inclined to treat the patients if they were older?**		
Yes	41	72%
No	16	28%
**Age Threshold for treatment (amongst physicians who were less inclined to treat older patients, n = 41)**		
65–74 years old	8	20%
75–84 years old	14	34%
85 years old and above	19	46%

The most important single factor that affected physicians’ decision for treatment of a geriatric oncology patient was performance status (53%), type of cancer (23%) and patient’s decision (11%). When asked to list the top 5 factors, the most important factors that emerged were performance status (95%), co-morbidities (75%), stage of cancer (75%), type of cancer (75%), patient’s decision (53%), and age (51%). Of note, slightly more than half the participants reported age as one of the top 5 factors that affected their treatment decision, although only 5% listed it as the most important single reason. Only 19% of cancer physicians ranked patient’s cognitive function in the top 5 important factors in determining cancer treatment. The majority (72%) of participants agreed that they would be less inclined to treat the patients if they were older. Amongst these participants, 80% reported the age threshold for less intensive treatment to be 75 years and above. There was no correlation between physician’s age (<=40 years versus >40 years) and reluctance to treat an older patient (77% vs 70%, p = 0.740). A substantial 37% of the physicians felt that our elderly patient population was often under-treated, and these physicians indicated the top 3 reasons to be patient’s preference (81%), family’s preference (66%) and poor performance status (47%).

### Disclosure of cancer diagnosis to patients

With regards to disclosure of cancer diagnosis to older patients, it was notable that only 9% of participants chose to disclose the cancer diagnosis to the patients directly, while 54% of physicians chose to disclose the cancer diagnosis to family members first. When asked to choose the top 3 reasons for non-disclosure, the reasons chosen were: the desire to comply with the family’ wishes for non-disclosure to the patient (84%), physician’s concern about patient’s inability to accept the diagnosis (55%) and the inability to understand the diagnosis (42%). In contrast, more than half the participants (61%) would disclose the diagnosis of cancer to the younger patients directly. About one-third of participants would disclose the cancer diagnosis to the patient and family members together for both older and younger patients (37% and 35% respectively). Importantly, none of the physicians would choose to disclose the diagnosis to only the elderly patient’s family and not the patient.

### Engagement of geriatric specialty services by cancer physicians

Most participants (61%) have never engaged the help of a geriatrician in the decision making process for cancer treatment and only about half the participants (47%) were aware that there were geriatric oncology assessment scales available. However, the vast majority (90%) welcomed the introduction of a geriatric oncology service, although a proportion (28%) expressed doubts about its feasibility.

### Response to clinical scenarios (Table 3)

**Table 3:** Response to clinical scenarios

**Scenario 1: Stage IV Diffuse Large B-cell Non-Hodgkin’s Lymphoma (n = 55)**		
**Treatment options for the older patient**	**Number**	**Percentage**
R-CHOP^1^ × 6 + Intrathecal Methotrexate	26	47%
R-CVP^2^ × 6	21	38%
CHOP^3^ × 6	4	7%
Palliative radiotherapy	3	6%
Best Supportive Care	1	2%
**Treatment options for the younger patient**	**Number**	**Percentage**
RCHOP × 6 + Intrathecal Methotrexate	45	82%
R-CVP × 6	7	13%
CHOP × 6	3	5%
Palliative radiotherapy	0	0%
Best Supportive Care	0	0%
Factors affecting treatment decision		
Performance Status	47	85%
Cancer type	37	67%
Cancer stage	20	36%
Patient decision	16	29%
Co-morbidities	15	27%
Age	14	25%
**Scenario 2: Stage IIB, Node positive, Hormone receptor positive Breast cancer (n = 53)**		
**Treatment options for the older patient**	**Number**	**Percentage**
Aromatase inhibitor × 5 years	34	64%
Tamoxifen × 5 years	8	15%
AC^4^ × 4 → T^5^ × 12, then endocrine treatment	5	9%
CMF^6^ × 6, then endocrine treatment	1	2%
FAC^7^ × 6, then endocrine treatment	1	2%
Others	4	8%
**Treatment options for younger patient**	**Number**	**Percentage**
AC × 4 → T × 12, then endocrine treatment	44	83%
FAC × 6, then endocrine treatment	5	9%
CMF × 6, then endocrine treatment	2	4%
Others	2	4%
Aromatase inhibitor × 5 years	0	0%
Tamoxifen × 5 years	0	0%
**Factors affecting treatment decision**	**Number**	**Percentage**
Cancer Stage	38	72%
Cancer Type	32	60%
Performance Status	25	47%
Age	24	45%
Patient decision	16	30%
Co-morbidities	9	17%

^1^R-CHOP: Rituximab, Cyclophosphamide, Doxorubicin, Vincristine and Prednisolone; ^2^R-CVP: Rituximab, Cyclophosphamide, Vincristine and Prednisolone; ^3^CHOP: Cyclophosphamide, Doxorubicin, Vincristine and Prednisolone; ^4^ AC: Doxorubicin and Cyclophosphamide; ^5^ T: Paclitaxel; ^6^CMF: Cyclophosphamide, Methotrexate and 5 Fluorouracil; ^7^FAC: 5 Flurouracil, Doxorubicin and Cyclophosphamide.

Two and four surveys each were incomplete for scenario 1 and scenario 2. Four participants who were haematologists were not familiar with treatment of breast cancer and opted not to fill in the section of the survey pertaining to the case scenario for breast cancer. Similarly, two oncologists opted not to fill in the lymphoma case scenario as they had limited experience treating patients with lymphoma.

In the first clinical scenario involving a fit elderly patient with stage IV diffuse large B cell non-Hodgkin’s lymphoma with an International Prognostic Index of 3, only 47% of participants chose the standard treatment of 6 cycles of R-CHOP or 2 cycles beyond best response with intrathecal methotrexate. In comparison, 82% of participants chose the same standard treatment for the younger patient.

In the second clinical scenario involving an elderly patient with stage IIB, hormone receptor positive, high risk node positive breast cancer, only 9% of physicians chose the option of adjuvant combined chemotherapy and hormonal therapy with 4 cycles of doxorubicin/cyclophosphamide, followed by weekly paclitaxel and an aromatase inhibitor for 5 years, which was the most aggressive treatment option. Almost 80% physicians chose only adjuvant endocrine therapy with an aromatase inhibitor (64%) or tamoxifen (15%) for 5 years without adjuvant chemotherapy. In contrast, if the patient was younger, at least 96% of participants would treat the patient with combined adjuvant chemotherapy and endocrine therapy and none would treat the patient with adjuvant endocrine therapy alone. Slightly more than two-thirds (68%) of participants would treat the elderly patient with adjuvant radiation therapy after lumpectomy surgery.

In both scenarios, physicians were asked to choose the 3 top factors that affected their decision for treatment. For scenario 1, most physicians chose performance status (85%), cancer type (67%) and cancer stage (36%) as the top 3 factors, while for scenario 2, the top 3 factors were cancer stage (72%), cancer type (60%) and performance status (47%).

## Discussion

Treatment of geriatric patients (>65 years old) with cancers can be challenging due to multiple medical, physiological, social as well as economic factors. In multi-ethnic Singapore, further challenges may arise from cultural differences, as well as the family’s involvement in disclosure of cancer diagnosis and formulation of treatment options.

In this study, physicians ranked age as only the sixth most important factor affecting their treatment decision for cancer patients, with only 5% of participants ranking it as the most important factor. The top factors chosen by the physicians were instead, performance status, co-morbidities, patient’s decision, cancer type and stage. Age was similarly not cited as one of the top three factors affecting treatment decisions in the two clinical scenarios. Yet, a striking discrepancy was observed in the actual treatment choices in the clinical scenarios, with most physicians opting for less intensive treatment for the older compared to the younger patient. The majority of the physicians also admitted that they were less inclined to treat patients more than 75 years old. These findings underscore the fact that advanced chronological age alone still deters most physicians from offering geriatric patients standard-of-care treatments in real life.

The geriatric population is a heterogeneous one with varying functional reserves and life expectancies. Particularly in this population, preserving functional status is as important an outcome as cancer-free survival. Anti-cancer treatments that are proposed must be in line with each patient’s life expectancy and performance status so that the patient is able to tolerate the side effects of the prescribed therapy and live long enough to derive actual efficacy benefits. Comprehensive geriatric assessment serves to define an initial level of performance, and yields information that may help guide physician decision about treatment after taking into account projected life-expectancy and potential tolerance to side-effects. Hence, a more structured approach is desirable to optimize treatment options for geriatric patients. Geriatric assessment scales have been developed to standardize and facilitate the decision making process with the help of a multi-disciplinary team, [13-15] and various studies have been done to evaluate the feasibility and practical application of these assessment scales in the healthcare setting [16-20]. For example, a study done in Singapore [16] looked at the incorporation of a Comprehensive Geriatric Assessment (CGA) scale in the management of Asian elderly cancer patients, and identified age, poor performance status, abnormal albumin level, abnormal geriatric depression scale, high malnutrition risk and advanced disease stage as independent predictors of overall survival. The integration of these assessment scales into routine oncology care may help to optimize care.

One potential barrier to managing an elderly cancer patient in Asia is the reluctance to disclose cancer diagnosis to the patient by family members. Singapore is a multi-ethnic country comprising of three major Asian ethnic groups, Chinese, Malay and Indian, that have different mother tongues, religious beliefs and cultural practices. In such a setting, the management of geriatric patients may pose an even greater challenge especially during communication. Amongst the Singaporean geriatric population, the majority (80%) have below secondary educational qualifications, and many spoke only their mother tongue (Chinese dialects, Malay, or Tamil) which the treating physician may not be fluent at, instead of the official language, English. As in most Asian countries, a large proportion (86%) stay with their spouse or children and the main source of financial support come from their children (63%) [28]. These social circumstances account for the important role the family plays during the decision making process for the treatment of elderly cancer patients.

Less than 10% of cancer physicians in this study chose to disclose cancer diagnosis to the older patients directly, in contrast to the preference of disclosing the news directly to the younger patient, though none of the physicians will opt to keep the diagnosis from the patients entirely. This is in contrast to the West, where disclosure of cancer diagnosis to the patient has been the norm for more than three decades [29-32]. A study by Novack *et al.*[29] in 1971 reported that 97% of physicians will disclose the truth to the cancer patients. In contrast, Kawakami *et al.*[33] showed that even with decisional ability, 15% of cancer patients in Japan were not told their cancer diagnosis as the wishes of their family governed whether disclosure occurred, although another Japanese study by Matsumura *et al.* in 1997 [34] showed that the majority of patients with cancer wished to know their diagnosis, regardless of the cancer stage. Some physicians may argue that withholding the diagnosis from elderly patients who do not wish to be told is a compassionate option that respects the patient’s wishes. Interestingly, two recent studies [35,36] showed that in patients with advanced cancer, there was in fact better emotional and quality of life with lack of awareness in patients. However, for patients who wish to know, it will be unethical to withhold the diagnosis. Some physicians attempt to reach a compromise by breaking the news in a sequential fashion to the patient and family, which allows for mutual emotional support and a combined discussion to facilitate decision making about treatment. This could be a feasible option in our cultural settings and was indeed the preferred option by one-third of the physicians we surveyed.

More than half of the participants in our study have never engaged the help of a geriatrician and half of them were not aware of the availability of geriatric assessment scales. 28% of the physicians were also concerned about the feasibility of establishing a geriatric oncology program in Singapore mainly due to the lack of geriatricians in Singapore. Reassuringly, the vast majority was receptive to the establishment of a formal geriatric oncology program. This may start with the integration of a structured geriatric assessment scale in routine oncology care. The current limitations of setting up a geriatric oncology program include the paucity of trained specialists such as geriatric oncologists and allied health staff. Nevertheless, these are changes that should be implemented with time in order to facilitate the management of the ageing population with cancer.

There are some limitations to this study. Firstly, the number of physicians who participated in the survey was less than half of the targeted population. Secondly, many of our participants were younger physicians, with only about a third of our participants having 6 years or more of clinical experience, although the majority had adequate exposure to cancer patients of the geriatric age group. Thirdly, our target cancer physician population included haematologists and medical oncologists some of whom were not familiar with the treatment of breast cancer and lymphoma patients respectively, resulting in incomplete responses to the two clinical scenarios.

## Conclusion

In Singapore, advanced age influenced treatment decisions among many cancer physicians, who are still inclined to treat an elderly cancer patient less intensively. Few cancer physicians were aware of formal geriatric assessment scales, and few have engaged the help of a geriatrician in the cancer treatment decision making process. However, most physicians were positive that a geriatric oncology program could benefit patients, and additional study to understand the attitudes of our geriatricians will be helpful as they will play an essential role in the development of a geriatric oncology program in Singapore. The practice of non-disclosure of cancer diagnosis is a potential barrier to the optimal management of geriatric patients, although in our cultural setting, disclosing the diagnosis to both the patient and family together or in a sequential manner could be a feasible compromise. The incorporation of assessment scales and management guidelines followed by establishment of a formal geriatric oncology program can potentially optimize the care of the elderly cancer patient in Singapore.

## Competing interests

The authors declare that they have no competing interests.

## Authors’ contributions

AP conceived the study, and participated in its design and coordination and helped to draft the manuscript. SH participated in the design and the execution of the study. SL was involved in overseeing the conceptualization of the study, execution, analysis of the data and writing of the manuscript. All authors read and approved the final manuscript.

## Pre-publication history

The pre-publication history for this paper can be accessed here:

http://www.biomedcentral.com/1471-2318/13/35/prepub

## Supplementary Material

Additional file 1 Questionnaire Survey on Cancer Physicians’ attitude towards treatment of cancers in geriatric patients. Click here for file
