# Supplementary material for: Cancer physicians’ attitude towards treatment of the elderly cancer patient in a developed Asian country
Source: BMC Geriatr. 2013 Apr 16;13:35. doi: 10.1186/1471-2318-13-35 (PMC3654995; doi:10.1186/1471-2318-13-35)
Supplement: Additional file 1 — Questionnaire Survey on Cancer Physicians’ attitude towards treatment of cancers in geriatric patients. [file 1471-2318-13-35-S1.doc]

**Additional files**

**A Questionnaire Survey on Cancer Physicians’ attitude towards treatment of cancers in geriatric patients.**

**Demographics**

**1**.Age: .

**2**.Gender: Male / Female

**3**.Occupation: Haematologist / Medical Oncologist / Radiation Oncologist

**4**.Institution (Work place): NUH / NCC/ SGH / Private Institutions: (name) .

**5**.No. of years in clinical practice as a cancer physician: ____________________________.

**6**.Average no. of patients seen per week: ________________________________________.

**7**.What is the estimated percentage of your patients who are >65 years old? : ___________.

------------------------------------------------------------------------------------------------------------

**Questions :**

**1**. Factors influencing your decision making process in the treatment of your patients >65y/o:

Rank the **Top 5 most important** factors (with 1 being the most important to 5 being the

least important)

**Factors: (15 choices)**

1. Age : ( )
2. Performance status : ( )
3. Highest level of education received ( )
4. Type of cancer : ( )
5. Stage of cancer : ( )
6. Presence of Co-morbidities : ( )
7. Psychological status (cognition) : ( )
8. Visual/Sensory Impairment ( )
9. Nutritional Status ( )
10. Financial Consideration ( )
11. Presence of a carer : ( )
12. Home environment : ( )
13. Patient being aware of the diagnosis ( )
14. Patient’s decision ( )
15. Family members’ decision ( )

**2**. For your geriatric patients in general, does it matter to you if your elderly cancer patients belong to the “young old”, “middle old” or “old old”? Yes / No

(** “young old”: 65 – 74 years old; “middle old”: 75 – 84 years old; “old old” >85 years old **)

**3**. Will you be less inclined to treat aggressively if your patient were older, even if he or she is physically fit with minimal co-morbidities? Yes / No

If Yes, What should be the “age threshold” for aggressive treatment in your opinion?

(A) “Young old” : 65 – 74 years old

(B) “Middle old”: 75 – 84 years old

(C) “Old old” : >85 years old

**4**. Do you feel that in Singapore, geriatric cancer patients are :

(A) Undertreated (B) Adequately treated (C) Overtreated (D) Don’t Know

If your answer is (A), why do you think is the reason? :

(Circle any of the following reasons, up to 3 choices)

(a) Physician preference

(b) Patient preference

(c) Family preference

(d) Geriatric patients tend to have multiple co-morbidities

(e) Geriatric patients have poorer performance status

(f) Geriatric patients have poorer physiological reserves

(g) Geriatric patients have poorer psychosocial support

**5**. In your older patient with cancer, how will you break the news to the patient and his/her family?

(A) Patient first, then family (B) Family members first, then patient (C) Only to the patient

(D) Only to the family members (E) To family and patient together

If your answer is (B) or (D), what will be your reason for doing so? :

(Circle any of the following reasons, up to 3 choices)

(a) Concern that the patient may not be able to accept the news.

(b) Concern that the patient may not be able to understand the condition well.

(c) Family may not want the patient to know

(d) Language or communication barriers

**6**. For a younger patient (50 years old) with cancer, how will you then break the news to the patient and his/her family? (Circle one of the following answers)

(A) Patient first, then family (B) Family members first, then patient (C) Only to the patient

(D) Only to the family members (E) To family and patient together

If your answer is (B) or (D), what is your reason for doing so? :

(Choose one of the following reasons, up to 3 choices)

(a) Concern that the patient may not be able to accept the news.

(b) Concern that the patient may not be able to understand the condition well.

(c) Family may not want the patient to know

(d) Language or communication barriers

**7**. Do you in your oncological practice, seek the help of a geriatrician or a psychogeriatrician to assess your patients’ level of fitness or cognition before deciding on treatment for geriatric patients with cancer?

(A) Never (B) Sometimes (C) Most of the time (D) Always.

**8**.Have you heard of any comprehensive geriatric assessment scales? Yes / No

If yes, please name those that you are aware of: _____________________________________.

**9**. Do you think it’s feasible to adopt a multidisciplinary approach (including geriatricians and allied health staff such as therapists) to the management of elderly patients with cancer in Singapore? Yes / No

If No, why? (Circle any of the following choices, up to 3)

1. Lack of awareness
2. Insufficient Geriatric physicians
3. Insufficient trained allied health staff
4. Too time-consuming
5. The feasibility is institution dependent
6. May delay management.

**10**. Do you think that the formal introduction of a Geriatric Oncology program in Singapore will allow for better management of elderly patients with cancer? Yes / No

If No, why? (Circle any of the following choices, up to 3)

1. Not feasible
2. May not be readily accepted
3. Too time consuming
4. Not likely to change outcome
5. Not yet. Will need time for people to be more aware of such a program
6. Just the primary physician and patient discussion will be sufficient in the decision making process for treatment.

**11**. Will you actively engage the help of the Geriatric Oncology team if it were to be introduced in Singapore? Yes / No

**Clinical Scenarios**

Do take some time to read the following 2 scenarios and answer the questions based on each of them.

**Case 1: Non-Hodgkin’s Lymphoma**

The patient is an 85 Chinese female who volunteers in the regional library. She had presented with a left neck lump that is increasing in size for the last 3 months and is now causing pain. She also had symptoms of chills and occasional fever. Further work-up with imaging and excision biopsy of the cervical lymph nodes revealed the diagnosis of Stage IV Diffuse large B cell lymphoma with bone marrow infiltration, IPI 3. Her transthoracic echocardiogram was normal with an ejection fraction of 65%. Other than hyperlipidemia and previous cataract surgery, she had been healthy for most of her life and was still actively looking after her grandchildren. Her ECOG performance status score is 1. Her husband, who accompanied her to the follow up visit, is a retired general practitioner and asks you several questions on the varying methods of treatment.

**1**. What form of treatment would you recommend to this patient (Circle only one answer)

(a) Rituximab + CHOP x 6 or 2 beyond best response + intrathecal methotraxate

(b) Rituximab + CVP x 6 or 2 beyond best response (omitting doxorubicin)

(c) CHOP x 6 or 2 beyond best response

(d) Only palliative RT to the symptomatic cervical lymph nodes.

(e) Best Supportive care and hospice referral.

**2**. What factors in this case most significantly influence how you would treat this patient?

(Choose from this list, up to 3 factors)

1. Age ( )
2. Performance status : ( )
3. Type of cancer : ( )
4. Stage of cancer : ( )
5. Presence of Co-morbidities : ( )
6. Psychological status (cognition) : ( )
7. Presence of a carer : ( )
8. Home environment : ( )
9. Patient being aware of the diagnosis ( )
10. Patient’s decision ( )
11. Family members’ decision ( )

**3**. What will be your treatment recommendation if this patient is 62 years old instead?

(Circle only one answer)

(a) Rituximab + CHOP x 6 or 2 beyond best response + intrathecal methotraxate

(b) Rituximab + CVP x 6 or 2 beyond best response (omitting doxorubicin)

(c) CHOP x 6 or 2 beyond best response

(d) Only palliative RT to the symptomatic cervical lymph nodes.

(e) Best Supportive care and hospice referral.

**Case 2: Early Breast Cancer**

An 85 year-old Chinese woman presented with a 2-cm breast mass, detected on a mammogram. On biopsy, it was proven to be a high grade, ER+ve/PR+ve HER-2-ve invasive ductal carcinoma. The patient prefers breast conservation therapy, but is open to all other treatment options. She undergoes a wide local excision of the breast tumour with axillary clearance. She had 2/10 LNs positive and her breast cancer is staged as IIB.

The patient lives with her daughter and family who are very supportive. She is very health conscious, and exercises everyday. She is very fit for her age (ECOG score, 0). She has no history of heart disease, diabetes mellitus, or hypertension. She only takes calcium and vitamin D supplements. Her ejection fraction is 68%.

**1**. Would you recommend radiation treatment for this patient?

(a)Yes

(b)No

**2**. Which of the following adjuvant regimens would you consider most appropriate for this patient?

(Circle only one answer)

(a) Aromatase inhibitors for 5 years

(b) Tamoxifen for 5 years

(c) CMF x 6, followed by endocrine therapy

(d) FAC x 6, followed by endocrine therapy

(e) AC x 4 -> Paclitaxel x 12, followed by endocrine therapy

(f) Others: _______________________________.

**3**. What factors in this case most significantly influence how you would treat this patient?

(Choose from this list, up to 3 factors)

1. Age ( )
2. Performance status : ( )
3. Type of cancer : ( )
4. Stage of cancer : ( )
5. Presence of Co-morbidities : ( )
6. Psychological status (cognition) : ( )
7. Presence of a carer : ( )
8. Home environment : ( )
9. Patient being aware of the diagnosis ( )
10. Patient’s decision ( )
11. Family members’ decision ( )

**4**. What will be your treatment recommendation if this patient is 60 years old instead?

(Select only one)

(a) Aromatase inhibitors for 5 years

(b) Tamoxifen for 5 years

(c) CMF x 6, followed by endocrine therapy

(d) FAC x 6, followed by endocrine therapy

(e) AC x 4 -> Paclitaxel x 12, followed by endocrine therapy

(f) Others: _______________________________.

------------------------------------------------------------------------------------------------------------------

Thank you for your participation.
